# Molecular evolution of aspartic protease gene family in vertebrates

**DOI:** 10.1038/s41598-026-52723-0

**Published:** 2026-05-20

**Authors:** Tatsuki Nagasawa, Tomofumi Inokuchi

**Affiliations:** 1 School of Life Science and Technology, Institute of Science Tokyo, Meguro-Ku, Tokyo, 152-8550 Japan; 2 Department of Biology, Cooperative Faculty of Education, Utsunomiya University, 350 Mine, Utsunomiya, Tochigi, 321-8505 Japan

**Keywords:** Aspartic protease, Pepsinogen, Gene family evolution, Gene loss, Vertebrates, Evolution, Genetics, Molecular biology, Zoology

## Abstract

Aspartic proteases are a gene superfamily with diverse functions in vertebrates, including pepsinogens (Pgs), the precursors of the major gastric enzyme pepsin. Despite their physiological importance, the evolutionary history of this gene family has been studied only in a fragmented manner. Here, we conducted comprehensive phylogenetic and genomic synteny analyses using whole-genome data from 75 vertebrate species to systematically elucidate the molecular evolution of aspartic protease genes. We identified *Pg* genes in cartilaginous fishes (elasmobranchs) for the first time, demonstrating that *Pg*s originated in the gnathostome ancestor. Pregnancy-associated glycoprotein (*PAG*) genes showed explosive expansion in cetartiodactyla but underwent secondary reduction in cetaceans. Cathepsin E (*ctse*), predicted to be the ancestral gene of *Pg*s, was also found in cartilaginous fishes and non-teleost ray-finned fishes (e.g., gar, bichir, bowfin), but was independently lost in multiple lineages, including teleosts, ruminants, vampire bats, and several stomach-less species. Our results reveal complex patterns of lineage-specific tandem gene duplication, lineage-specific expansion, and convergent gene loss, providing an evolutionary framework for understanding the functional diversification of aspartic proteases in relation to feeding strategies, digestive physiology, and reproductive adaptations in vertebrates.

**Supplementary Information:**

The online version contains supplementary material available at 10.1038/s41598-026-52723-0.

## Introduction

Aspartic proteases constitute a class of proteolytic enzymes that exhibit catalytic activity under acidic conditions. They characteristically cleave peptide bonds through hydrolysis using two conserved aspartic acid residues located in the active site, from which their name is derived^1,2^. Members of this gene superfamily are broadly distributed across vertebrates^3^, plants^4^, fungi^5^, and various bacteria and viruses^2^, and they perform diverse physiological roles across tissues.

Among vertebrate aspartic proteases, pepsinogens (Pgs)—the precursors of pepsins, the major gastric digestive enzymes and members of the peptidase A1 family—represent the most thoroughly investigated group^3,6^. Research on pepsin has a long history: following its naming by Theodor Schwann in 1836^7,8^, pig pepsin was crystallized in 1929^9^, and its amino acid sequence was later determined^10^. Subsequent studies across many vertebrate taxa rapidly expanded pepsinogen sequence data^3^. Comparative genomic analyses revealed that pepsinogens form a multi-copy gene family in several vertebrates and can be broadly divided into type-A and type-C lineages^3,11^. Type-A pepsinogens comprise several subfamilies. In addition to PgA, the major pepsinogen in humans and many other vertebrates, this group includes chymosin (cym)—also known as rennin—the key component of rennet synthesized and secreted by chief cells of newborn mammals to coagulate milk^12^. It further includes pepsinogen F (*pgf*), which functions in both the neonatal stomach and the placenta^3,13^, as well as the pregnancy-associated glycoproteins (PAGs). Although PAGs are phylogenetically close to *pgf*, they are not expressed in the stomach but instead show placenta-specific expression and are widely used as pregnancy markers in cattle and other mammals^14,15^. With the availability of whole-genome sequences, type-C pepsinogens have further been subdivided into PgC (progastricsin), PgB, mammalian PgC1, amphibian PgBC, and other lineage-specific paralogs^3,16^. Thus, in addition to canonical gastric Pgs expressed and secreted by chief cells, a number of functionally divergent subfamilies have also diversified throughout vertebrate evolution.

Vertebrates also possess multiple additional aspartic proteases that share sequence similarity with pepsins and belong to the peptidase A1 family. These include renin (*ren*), secreted from the kidney and involved in blood pressure regulation through angiotensinogen processing^17^; napsin A (*napsa*), expressed in lung epithelial cells and used as a diagnostic marker for adenocarcinoma^18,19^; cathepsin D (*ctsd*), a lysosomal enzyme expressed in various tissues^20^; and cathepsin E (*ctse*), originally characterized as a “pepsin-like endopeptidase” enriched particularly in the stomach^21,22^. The estrogen-responsive nothepsin (*nots*), expressed in female livers^23^, represents yet another example. In addition, the peptidase A22 family contains membrane-anchored aspartic proteases such as β-secretase-1 (*bace1*), which contributes to amyloid-β production and has been implicated in Alzheimer’s disease^24,25^, and its paralog *bace2*^26^. Collectively, these genes illustrate the extensive diversification of aspartic proteases in vertebrates and highlight the complex evolutionary processes that have shaped this family.

Several previous studies have discussed the evolutionary origin of the aspartic protease family in vertebrates. Some members, such as *ctsd* and *bace1/bace2*, have evolutionary origins that predate vertebrates. For example, *ctsd* has been identified in Aspergillus^27^ and insects^28^, while *bace1* and *bace2* have been reported in cnidarians^29,30^. In contrast, *napsa*^31^ and *nots*^23^ are present in both tetrapods and teleosts, suggesting an origin that can be traced back at least to the common ancestor of osteichthyans. *Ctse* has also been reported in amphibians^32–36^ and, more recently, in teleosts such as bitterling^37^. Pepsinogens (Pgs) are thought to have originated from the common ancestor of ctse and Pgs^3^. Although the presence of Pg-like activity has been suggested in sharks based on biochemical evidence^38^, Pgs are absent from the full genome sequence of the stomach-less chimaera *Callorhinchus milii*^39^, leaving their evolutionary origin unresolved. Chymosin (cym) and pga constitute a closely related sister-group pair found exclusively in amniotes; however, both genes have independently become pseudogenized in several mammalian lineages, including primates and perissodactyls^3,40,41^. Although considerable knowledge has accumulated regarding individual members of the vertebrate aspartic protease family, most evolutionary inferences have been based on limited taxa sampling or partial sequence information^3^. As a result, a comprehensive and systematic understanding of the diversification processes that shaped the entire family across vertebrates is still lacking.

With the recent expansion of high-quality vertebrate genome assemblies, it has become possible to trace the evolutionary history of large multigene families in unprecedented detail. This development enables more precise inference of the evolutionary timing of gene gains and losses that have previously been discussed based on limited taxonomic sampling. In particular, the increasing availability of genome data from cartilaginous fishes and non-teleost ray-finned fishes (e.g., gar, bichir, bowfin) provides a critical evolutionary bridge between earlier discussions centered primarily on tetrapods and teleosts. In this study, we focus particularly on the origin and diversification of pepsinogens and integrate molecular phylogenetic analyses with genomic synteny comparisons across 75 representative vertebrate genomes. As a result, we provide the first comprehensive and systematic reconstruction of the evolutionary trajectories of the aspartic protease family in vertebrates.

## Results

### Evolution of aspartic proteases

**Fig. 1:**
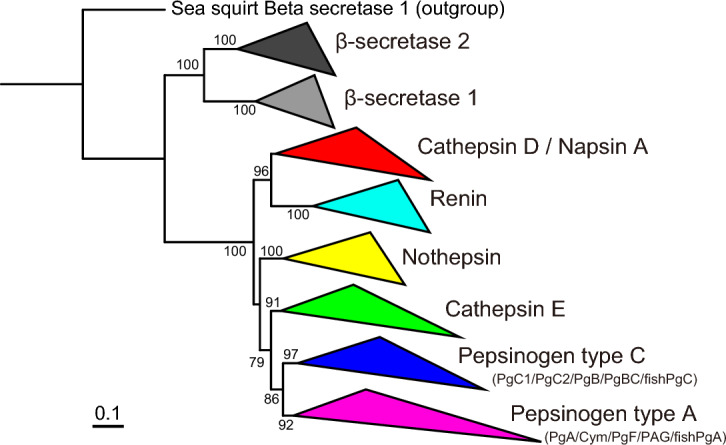
Phylogenetic analysis of all aspartic proteases in vertebrates. Maximum-likelihood tree reconstructed from 897 aspartic protease sequences identified from 75 vertebrate genomes. The tree was rooted using the β-secretase (*bace*) gene from the ascidian *Ciona intestinalis* as an outgroup. Each major subfamily is collapsed to facilitate visualization of higher-level relationships. Bootstrap support values (100 replicates) are shown at major nodes; only values ≥ 50% are indicated. The fully expanded tree including all sequences and bootstrap values is provided in Fig. S1.

To obtain an overview of the evolutionary history of the vertebrate aspartic protease family, we first selected 75 representative species across major vertebrate lineages (Table S1). From their whole-genome assemblies, we retrieved all candidate aspartic protease genes and identified a total of 897 sequences through phylogenetic inference (Fig. 1, Fig. S1). Consistent with previous studies^32^, vertebrate aspartic proteases were resolved into the following major clades: β-secretase 1 (bace1), β-secretase 2 (bace2), Nothepsin (nots), Renin (ren), Cathepsin D/Napsin A (ctsd/napsa), Cathepsin E (ctse), and Pepsinogen type-A/-C (Fig. 1). Most clades were strongly supported as monophyletic by high bootstrap values, with the exception of the *ctsd* and *napsa* groups, which exhibited substantial sequence similarity and were not clearly separable in the phylogeny (Fig. S1). Because sequence-based phylogenetic analyses did not clearly resolve *ctsd* and *napsa*, we distinguished these two genes based on their genomic synteny (Fig. S2, S3). Whereas the genomic neighborhoods of *napsa* differed among tetrapods, teleosts, and amphibians (Fig. S3), the synteny surrounding *ctsd* was highly conserved across vertebrates (Fig. S2), allowing unambiguous discrimination between the two. As described in later sections, the remaining aspartic protease families also exhibited largely conserved genomic contexts, which were consistent with the phylogenetic placements and facilitated accurate annotation (Fig. S4– Fig. S8). Among these genes, *nots*, *ren*, *ctse*, and pepsinogen type-A/-C were present in cartilaginous fishes, suggesting their origin in the common ancestor of jawed vertebrates. In contrast, *napsa*, *ctsd*, and *bace1* were also detected in the genomes of jawless vertebrates, indicating that these genes were already present in the last common ancestor of extant vertebrates (Table S1, Fig. 1).

### Evolution of pepsinogens

**Fig. 2:**
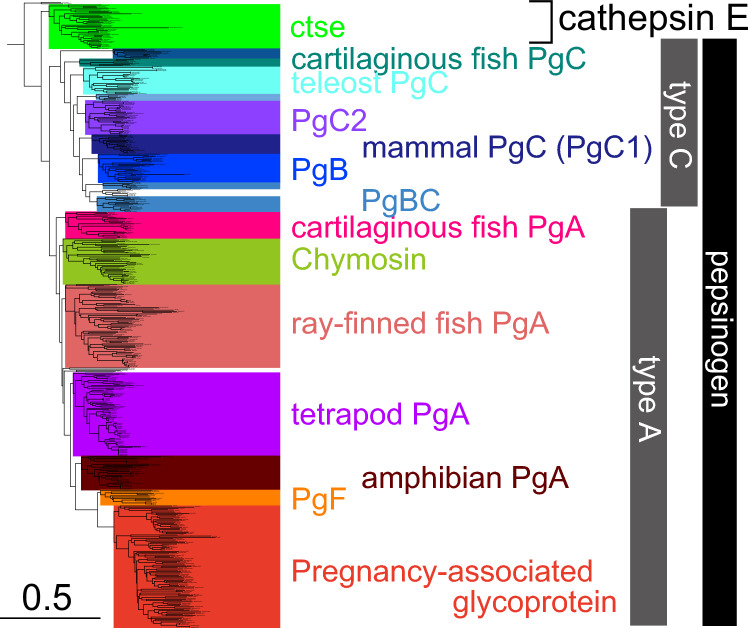
Phylogenetic analysis of ctse and pepsinogens. Maximum-likelihood tree of all *ctse* and pepsinogen genes used in this study. This figure highlights relationships among major pepsinogen subfamilies. The fully expanded phylogenetic tree is provided in Supplementary Fig. S9.

To investigate the evolutionary history of individual pepsinogen (Pgs) subfamilies, we next performed a phylogenetic analysis using *ctse*—the closest outgroup to Pgs—based on our comprehensive survey (Fig. 2, Fig. S9). Overall, the resulting topology was highly consistent with previous findings^16,39^: pepsinogen type-C segregated into PgC1, PgC2, PgB, PgBC, and teleost PgC, while pepsinogen type-A formed major clades corresponding to ray-finned fish PgA, tetrapod PgA, amphibian PgA, cymosin, PgF, and pregnancy-associated glycoproteins (PAG). In the following sections, we describe the detailed evolutionary trajectories of each subfamily by integrating these phylogenetic relationships with comparative genomic synteny analyses.

### Evolution of pepsinogen type-C

**Fig. 3:**
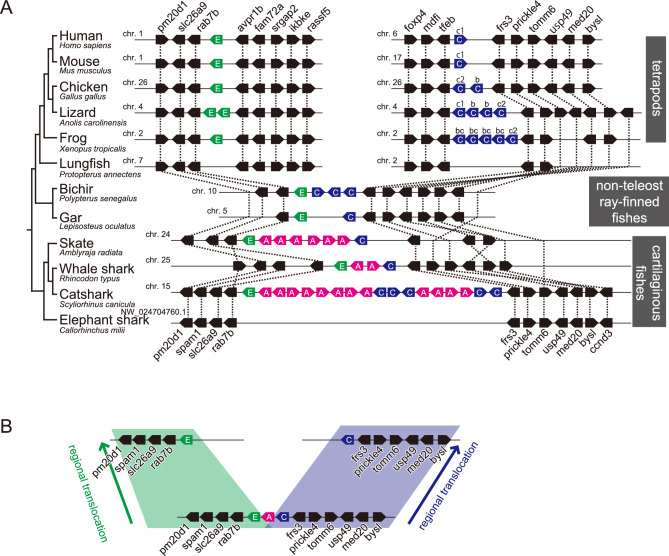
Evolutionary process of *ctse* and pepsinogens through tandem gene duplication and regional translocation. (**A**) Genomic synteny of *ctse* and pepsinogens. Genes and their transcriptional orientation are indicated by pentagons, with *ctse*, *pga*, and *pgc* highlighted in green (“E”), red (“A”), and blue (“C”), respectively. Phylogenetic relationships of species are shown on the left, and species names follow Table S1. Homologous genes are connected by dotted lines. (**B**) Schematic diagram illustrating regional translocation of *ctse* and *pgc*.

In cartilaginous fishes, *ctse*, *pga*, and *pgc* formed a compact gene cluster flanked by *rab7b* and *frs3* (Fig. 3). Specifically, these genes were arranged in tandem, comprising four genes within 157 kb in whale shark *Rhincodon typus*, eight genes within 313 kb in skate *Raja radiata*, and 18 genes within 580 kb in catshark *Scyliorhinus canicula* (Table S2). This conserved genomic arrangement was not unique to cartilaginous fishes; rather, a similar synteny pattern was also observed in non-teleost ray-finned fishes: bichir *Polypterus senegalus,* bowfin *Amia calva,* and gar *Lepisosteus oculatus* (except for *pga*, which had translocated to a different genomic region). These results suggested that *ctse*, *pga*, and *pgc* originated through tandem gene duplication events (Fig. 3A). The genomic synteny of both *ctse* and *pgc* was further conserved among tetrapods. In tetrapods, *rab7b* remained adjacent to *ctse*, whereas *frs3* was consistently located next to *pgc*, as in cartilaginous fishes and non-teleost ray-finned fishes. Although tetrapod *ctse* and *pgc* are positioned on different genomic regions (or chromosomes), each retains one side of the ancestral syntenic block found in cartilaginous and non-teleost ray-finned fishes, implying that regional translocation occurred during tetrapod evolution (Fig. 3B). As previously reported^16,39^, PgC subtypes (such as PgC1, PgBC, and PgC2) were typically arranged in tandem, and the synteny of all non-teleost pepsinogen type-C genes—including cartilaginous fish PgC—was highly conserved (Fig. 3). Teleost PgC represented the sole exception, occupying a distinct genomic location with its own highly conserved synteny pattern (Fig. S8). In addition, additional *pgc* loci were identified at separate genomic regions specifically in Otophysa (Fig. S8).

### Evolution of pepsinogen type-A

**Fig. 4:**
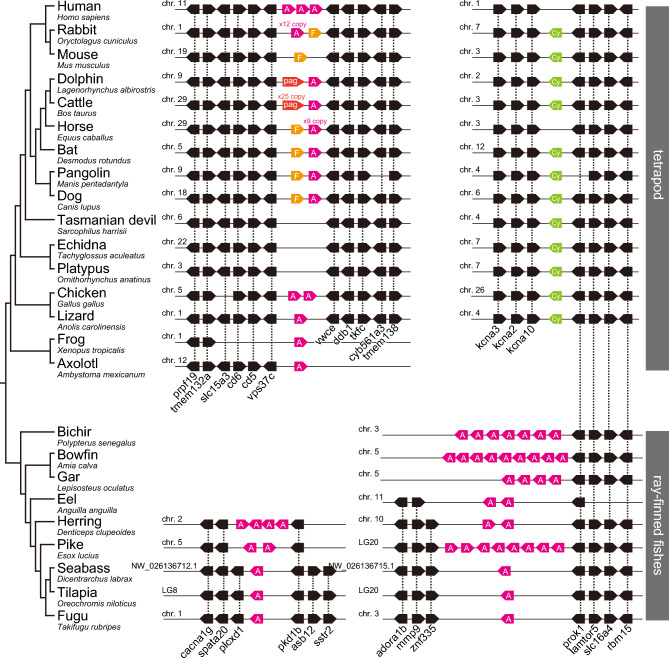
Genomic synteny of pepsinogen type-A. Genomic synteny is shown in the same way as in Fig. 3, with *pga*, *pgf*, *cym*, and *PAG* indicated in red (“A”), bright yellow (“F”), yellow-green (“Cy”), and orange (“pag”), respectively. Gene copy numbers are shown above genes with multiple copies.

**Fig. 5:**
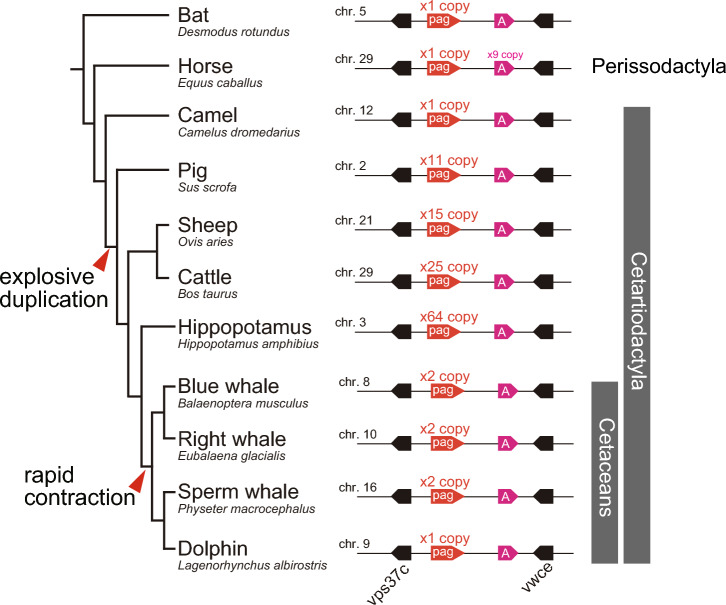
Evolutionary process of pregnancy-associated glycoproteins (PAGs). Genomic synteny is shown as in Fig. 3, with *pga* and *PAG* indicated in red (“A”) and orange (“pag”), respectively. Gene copy numbers are shown above genes with multiple copies. Evolutionary events that occurred in the *PAG* gene are indicated by red arrowheads.

**Fig. 6:**
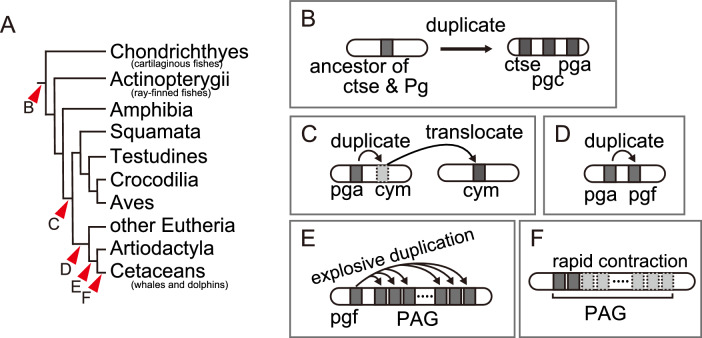
Evolutionary history of pepsinogen genes in vertebrates. (**A**) Timing of the evolutionary events of pepsinogens. (**B**) Tandem gene duplication of *ctse*, *pga* and *pgc* in the common ancestor of jawed vertebrates. (**C**) Gene duplication and translocation of *pga* and *cym* at the common ancestor of amniotes. (**D**) Tandem gene duplication of *pga* and *pgf* in the common ancestor of Eutheria. (**E**) Explosive gene duplication of *PAG* genes at the common ancestor of Cetartiodactyla. (**F**) Rapid gene reduction of *PAG* genes in whales.

The genomic synteny of pepsinogen type-A was generally well conserved across vertebrates (Fig. 4), except in cartilaginous fishes, where *pga*, *pgc*, and *ctse* formed a compact gene cluster. In contrast, the syntenic context of *pga* in ray-finned fishes did not correspond to that of tetrapod *pga*; instead, it closely resembled the genomic location of tetrapod *cym*. This pattern strongly supports the classic hypothesis that *cym* originated via gene duplication of *pga*^3^. In eutherian mammals, *pga* and *pgf* (or *PAG*) were located in tandem, with the exception of humans *Homo sapiens*, suggesting that *pgf/PAG* likely arose through tandem duplication of *pga*. Within Cetartiodactyla, the *pgf/PAG* genes underwent massive lineage-specific amplification (e.g., cattle *Bos taurus*: 25 copies; hippopotamus *Hippopotamus amphibius*: 64 copies), whereas most other eutherians—including cetaceans and perissodactyls—retained only one or two copies. These results indicate that Cetartiodactyla experienced an extensive expansion of *pgf/PAG*, followed by secondary contraction in cetaceans (Fig. 5). A summary of the evolutionary history of pepsinogens inferred in this study is shown in Fig. 6.

### Evolution of cathepsin E

**Fig. 7:**
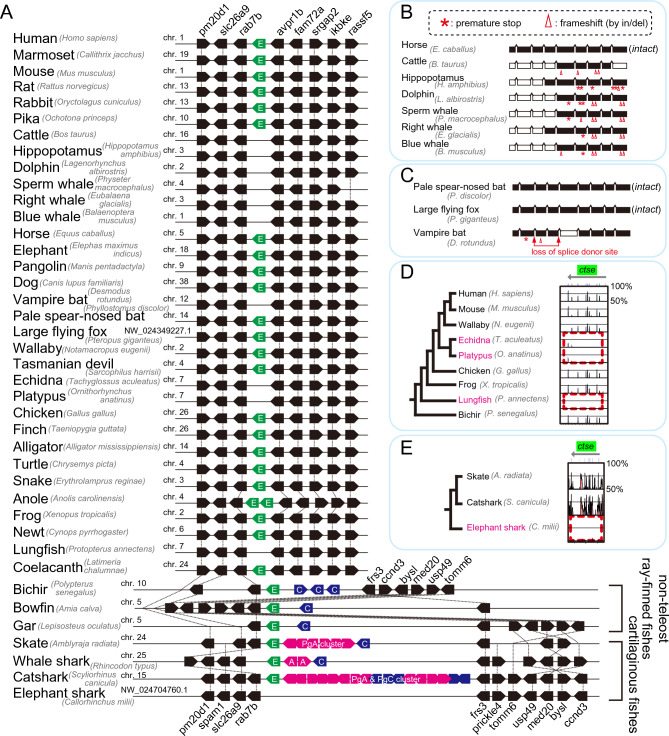
*Ctse* genes were highly conserved in jawed vertebrates, but convergently lost in some lineages. (**A**) Genomic synteny of ctse genes. Convergent gene loss is shown in (**B**) Cetartiodactyla, (**C**) vampire bat, (**D**) lungfish and monotremes, and (**E**) elephant shark. Genomic synteny is shown as in Fig. 3. (**B**–**C**) Schematic representation of mutations causing pseudogenization. (**D**–**E**) Partial VISTA plots showing broad-scale alignments. In monotremes, lungfish, and elephant shark, exon conservation (peaks) was nearly absent, indicating complete pseudogenization. Multiple sequence alignments of pseudogene fragments are shown in Fig. S10.

In this study, we demonstrate for the first time that both cartilaginous fishes and non-teleost ray-finned fishes retain the *ctse* gene (Fig. 7A). The genomic synteny of *ctse* was highly conserved throughout vertebrate evolution (Fig. 7A); however, several lineages exhibited secondary loss of this gene (Fig. 7B–E, Fig. S10). Consistent with previous reports, *ctse* was absent in cetartiodactyls (Fig. 7B)^42^, vampire bats (Fig. 7C)^43^, and monotremes (Fig. 7D)^40^. In addition, our analyses revealed that lungfish *Protopterus annectens* and elephant shark have also lost *ctse* (Fig. 7D and E). A previous study^37^ reported the presence of *ctse* in nine teleost species, including bitterling (*Rhodeus uyekii*), channel catfish (*Ictalurus punctatus*), and tilapia (*Oreochromis niloticus*). However, in our phylogenetic analyses, all teleost sequences previously annotated as *ctse* grouped within the Nots clade (Fig. S11A). Likewise, their genomic synteny patterns were consistent with *nots* rather than *ctse* (Fig. S11B). This discrepancy likely arose because the previous study^37^ did not include *nots* sequences in the phylogenetic reconstruction. Despite extensive searches across all available genomes, we did not detect any bona fide *ctse* genes in teleosts (Table S1; Fig. 1). Taken together, these findings indicate that teleosts have secondarily lost the *ctse* gene.

## Discussion

In this study, we conducted a comprehensive and systematic investigation of the molecular evolution of the vertebrate aspartic protease family, with particular emphasis on the pepsinogen (Pg) lineage. Our analyses revealed that nearly all members of this family originated through gene duplication, most notably tandem gene duplication. Although teleosts experienced a teleost-specific whole-genome duplication (3R) in their common ancestor, most duplicated gene pairs generated by this event are known to have been rapidly reduced to single-copy states during subsequent evolution^44^. Consistent with this general pattern, our analyses did not identify clear 3R-derived duplicate pairs within the teleost aspartic protease family (e.g., Fig. S8). This observation suggests that lineage-specific processes, such as tandem duplication and gene loss, have played a more prominent role in shaping the evolution of this gene family than teleost-specific whole-genome duplication itself. In addition to clarifying the evolutionary relationships and branching patterns among paralogous genes that had previously been inferred from a limited number of species, we uncovered lineage-specific expansions, losses, and chromosomal translocations, thus providing a more detailed view of the dynamic evolutionary history of this gene family (e.g. Fig. 6). For example, the neonatal milk-clotting enzyme chymosin (*cym*) and the major digestive enzyme pepsinogen A (*pga*)^3,12^ were shown to have clear orthologous relationships with pregnancy-associated glycoprotein (*PAG*)—essential for pregnancy maintenance and placental function in ruminants—and pepsinogen F (*pgf*), which is expressed during fetal development and contributes to neonatal digestion. Furthermore, by integrating molecular phylogenetics with genomic synteny analyses, we were able to precisely determine the evolutionary timing at which these genes arose. These findings provide an essential framework for interpreting the diversification of gene functions and expression profiles within this family.

### Reevaluating the evolutionary origins of aspartic protease genes

The recent availability of high-quality genomes from a wide range of vertebrate lineages—including so-called “ancient fishes” such as non-teleost ray-finned fishes and lobe-finned fishes, as well as cartilaginous fishes—has made it possible to place molecular evolutionary events within a broader, vertebrate-wide context. By leveraging these resources, our comparative genomic analyses enabled us to reassess the evolutionary origins of multiple aspartic protease genes. For the *napsa* gene, previous synteny-based analyses suggested that its origin dates back at least to the common ancestor of tetrapods^31^. However, our analyses demonstrated that, although the surrounding genomic architecture differs markedly among tetrapods, teleosts, and amphibians, the synteny around *napsa* is highly conserved in non-teleost ray-finned fishes and cartilaginous fishes, resembling that of tetrapods (Fig. S3). These findings suggest that the *napsa* genomic region has undergone independent chromosomal rearrangements in the teleost and amphibian lineages. Our study also uncovered new evolutionary insights into *nots*. This gene had previously been identified in teleosts and tetrapods^23^, and was therefore thought to have originated in the common ancestor of bony vertebrates. Here, we show for the first time that *nots* are also preserved in cartilaginous fishes (Fig. S6), indicating that its origin may date back to the common ancestor of gnathostomes—or potentially even earlier. Intriguingly, *nots* has been secondarily lost in mammals, with monotremes being the sole exception (Fig. S6 and Table S1). Although the physiological role of nots remains largely unknown, its ancient origin and lineage-specific loss patterns provide important clues for future functional studies.

### Origin of the jawed vertebrate digestive system and the emergence of pepsin-based digestion

The acquisition of jaws enabled vertebrates to ingest larger and more diverse prey^45,46^. In addition, the acquisition of a functional stomach enabled a digestive system capable of storing large meals for extended periods and efficiently degrading them under highly acidic conditions using potent proteolytic enzymes^47^. Given these innovations, the emergence of pepsinogens (Pgs)—the precursors of the major gastric protease pepsin—is expected to have occurred in the common ancestor of jawed vertebrates (Gnathostomata). Although biochemical studies suggested the presence of pepsin-like activity in the stomach of sharks^3,38^, no Pg genes were identified in the genome of the elephant shark (*Callorhinchus milii*), a holocephalan species lacking a stomach^39^. Because Pgs are repeatedly and convergently lost in stomach-less vertebrates^39,48^, it has remained unclear whether cartilaginous fishes retained Pgs or lost them secondarily. In this study, we newly identified Pgs genes in the genomes of elasmobranchs—whale shark *Rhincodon typus*, catshark *Scyliorhinus canicula*, and skate *Amblyraja radiata*—all of which possess a stomach (Fig. 2). These findings indicate that cartilaginous fishes share a pepsin-based digestive system with many other vertebrates, strongly supporting the view that Pgs originated in the last common ancestor of Gnathostomata.

### Copy number diversification of pepsinogens and its physiological implications

**Fig. 8:**
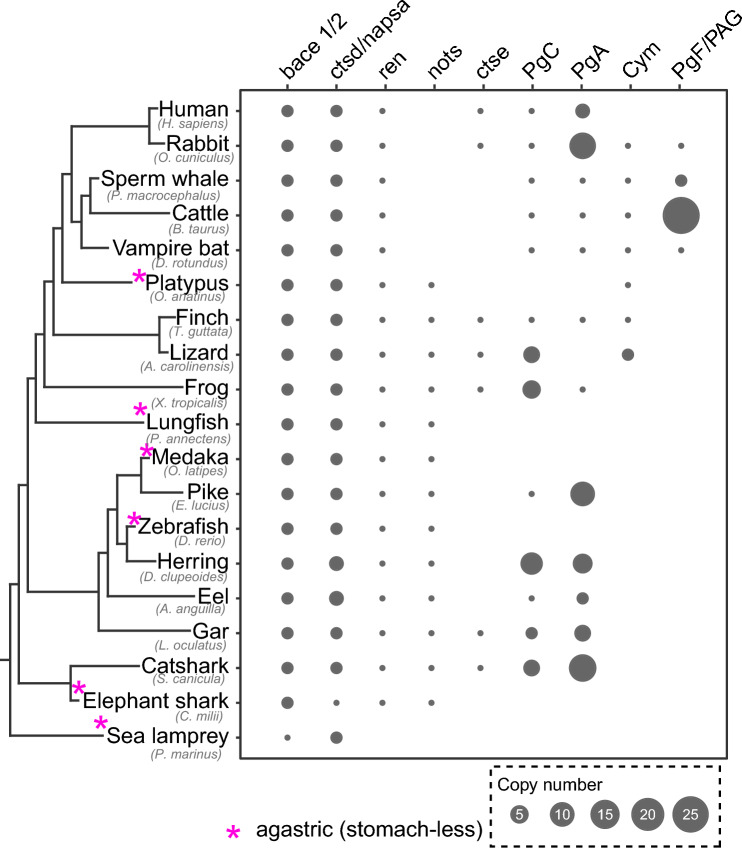
Copy number variation of aspartic protease genes across vertebrates. Bubble size indicates gene copy number. Magenta asterisks indicate agastric (stomach-less) species.

Our results also provide insight into the diversification of pepsinogen (Pg) copy numbers across vertebrates. As previously reported^39,48^, Pgs are consistently absent in agastric (stomach-less) species (Fig. 8; Table S1), reinforcing the tight functional association between Pgs and the gastric digestive system. In contrast to the generally stable copy numbers observed in other aspartic protease genes, Pgs exhibited lineage-specific expansions in several taxa (Fig. 8). Interestingly, increased Pg copy numbers were detected not only in carnivorous species such as pike *Esox lucius* and catshark, but also in herbivorous mammals such as rabbit *Oryctolagus cuniculus* (Fig. 8). This pattern suggests that Pg copy number expansion cannot be explained solely by dietary preference. Rather, digestive capacity mediated by Pgs is likely influenced by multiple factors, including gene expression levels, enzymatic activity, substrate specificity, and gastric physiology^49–51^. Therefore, simple correlations between Pg copy number and trophic ecology may not fully explain the observed evolutionary patterns. Taken together, these findings suggest that while the origin of Pgs was tightly linked to the emergence of a functional stomach in jawed vertebrates, subsequent changes in Pg copy number may reflect lineage-specific fine-tuning of digestive physiology rather than direct adaptation to broad dietary categories. Future integration of comparative genomics with physiological and biochemical data will be essential to clarify how gene copy variation translates into functional diversification within the vertebrate digestive system.

### Evolutionary history of cathepsin E

Pepsinogens (Pgs) are synthesized and secreted by chief cells or oxyntic–peptic cells in the gastric glands^52,53^. In addition to Pgs, the gastric mucosal epithelium expresses cathepsin E (*ctse*)^21,36,54,55^. Based on its monophyly in molecular phylogenies and its acidic optimal pH^56^, Pgs have long been hypothesized to have originated from *ctse*^3^. However, the presence of *ctse* in cartilaginous fishes has been largely unexplored. Here, we newly discovered *ctse* sequences in the genomes of elasmobranchs, whereas neither *ctse* nor Pgs were detected in any jawless vertebrate (Table S1). These results suggest that *ctse* was acquired in the common ancestor of Gnathostomata. Notably, we also show for the first time that non-teleost ray-finned fishes retain the *ctse* gene, whereas all analyzed teleosts lack it (Fig. 3). Whether teleosts possess *ctse* has long been a matter of debate. Borrelli et al*.* previously analyzed gene sequence data from 17 vertebrate species, including seven teleosts, but did not identify *ctse* in any teleost genome^57^. Subsequently, Kim et al*.* reported that nine teleost species possess *ctse* genes^37^; however, our phylogenetic and synteny analyses strongly support that all of these genes correspond instead to nothepsin (*nots*), a sister lineage of *ctse*. This confusion likely stems from the highly similar sequence characteristics between *ctse* and *nots*, as nots was historically referred to as a “cathepsin E-like protease” in chicken^23,58,59^ and was not included in the phylogenetic framework of the previous study^37^. Taken together, these findings demonstrate that *ctse* has been lost in the teleost lineage, consistent with the conclusions of Borrelli et al*.*^57^.

Beyond teleosts, we found that *ctse*, like Pgs, has been independently lost in multiple vertebrate lineages (Fig. 7)^40,42,43^. Stomach-less species such as the elephant shark (Holocephali), lungfish *Protopterus annectens* (Sarcopterygii), and monotremes (echidna *Tachyglossus aculeatus* and platypus *Ornithorhynchus anatinus*) lack both Pgs and *ctse*^39,40^. By contrast, ruminants—whose foregut fermentation involves partial pre-gastric digestion followed by regurgitation and rechewing—and blood-feeding vampire bats retain Pgs but have lost *ctse* (Fig. 7). At present, the only well-characterized physiological function of cathepsin E is antigen processing via the MHC class II pathway in mammalian antigen-presenting cells^55^. The repeated, lineage-specific losses of *ctse*, including its loss in teleosts, provide important insights into the physiological role of *ctse* in the vertebrate stomach.

### Discussion on pregnancy-associated glycoproteins

Pregnancy-associated glycoproteins (PAGs), widely used as pregnancy markers in ruminants^14,15^, are known to have arisen during the evolution of Cetartiodactyla^60^. Our analyses strongly support that pepsinogen F (*pgf*) and *PAG*s form a monophyletic group (Fig. 2). Although *pgf* and PAGs formed clearly distinct clades, we found that the horse gene previously described as PAG based on its expression and functional properties^14,61^ actually clusters within the *pgf* clade. These results suggest that distinguishing *pgf* from *PAG* solely on the basis of sequence characteristics is inherently challenging. We further showed that PAG genes underwent an explosive expansion in ruminants, whereas camelids (Camelidae) and cetaceans (Cetacea) retained only one or two copies. Although the function of PAGs remains unresolved, they are primarily expressed in binucleate trophoblast cells (BNCs) in ruminants^62,63^. Notably, placental observations in baleen whales (*Balaenoptera acutorostrata*, *B. brydei*, and *B. borealis*) report an absence of BNCs^64^. Therefore, it has been suggested that the secondary reduction of *PAG* genes in cetaceans may be linked to the evolutionary degeneration of BNCs in their placenta. In contrast, whether BNCs are present in camelids, which represent an early-diverging cetartiodactylan lineage, remains inconclusive^65,66^. Future studies integrating *PAG* copy-number variation, their expression profiles, and placenta morphology across Cetartiodactyla—including camelids—will be essential for elucidating the functional significance and evolutionary constraints shaping PAG evolution.

## Materials and methods

### Gene identification and sequence retrieval

Gene identification was conducted following previous study^67^, with methodological refinements as follows. We first retrieved annotated sequences for each gene family from the NCBI database. These sequences were then used as queries for BLAST searches against whole-genome assemblies available in NCBI to isolate additional homologs. For each genome assembly, we performed TBLASTN searches using amino acid sequences from closely related species to find candidate genomic regions. Exon–intron structures were subsequently inferred using GeneWise2^68^. All predicted coding sequences were subjected to multiple sequence alignment and manually inspected to ensure accurate sequence annotation. Accession numbers for all isolated genes and genome assemblies used in this study are listed in Table S1. The query sequences used for BLAST searches consisted of previously characterized and experimentally validated gene sequences, which are summarized in Table S3. The species selected for analysis represent the major vertebrate lineages. Importantly, the sampling also incorporates taxa with differences in stomach presence or absence, thereby enabling discussion of evolutionary patterns in relation to physiological traits such as pepsinogen-dependent digestion. In cases where lineage-specific gene expansions or contractions were detected, additional representative species were included to more precisely infer the evolutionary timing of these events. Gene copy numbers (e.g., *PAG1*, *PAG2*) were assigned according to their order within each genome assembly and do not necessarily reflect historical nomenclature.

### Molecular phylogenetic analysis

Amino acid sequences for each aspartic protease gene family were aligned using MAFFT (ver. 7)^69^. The best-fitting substitution models were identified using ModelTest-NG^70^. Phylogenetic reconstruction was then performed under the maximum-likelihood framework implemented in RAxML-NG^71^, with 100 bootstrap replicates to assess node support. Resulting trees were visualized and annotated using iTOL (ver. 6)^72^.

### Genomic synteny analysis

Comparative genomic synteny around each target gene was examined using Genomicus^73^ and the NCBI Genome Data Viewer^74^. To confirm orthology and evaluate local gene order conservation, candidate loci were further inspected through TBLASTN searches against the available genome assemblies for each species. Only gene relationships supported by both syntenic context and sequence similarity were retained as putative orthologs. Because each subfamily exhibits a complex evolutionary history, synteny results are presented across multiple figures organized by clade or genomic region. Consequently, some results appear in more than one figure for clarity.

### Visualization of the pseudogenes

Pseudogene candidates were identified using two complementary approaches. First, we examined conserved genomic regions with VISTA^75^ to visualize patterns of sequence degradation or loss. In particular, the genomic loci corresponding to the ctse fragments were assessed using multi-LAGAN alignments^76^, allowing us to compare the interval between syntenic flanking genes across species with high-quality genome assemblies. Only genomes in which both neighboring genes were located on the same scaffold or chromosome were included, ensuring reliable inspection of the intergenic regions. Second, to corroborate the presence of pseudogenized remnants, we performed TBLASTN searches against the available genome datasets from related taxa. Hits showing partial similarity, frameshifts, premature stop codons, or disrupted open reading frames were considered indicative of pseudogene status. Together, these analyses allowed us to pinpoint degraded gene fragments and evaluate their evolutionary conservation or lineage-specific loss.

## Conclusion

This study presents the comprehensive reconstruction of the evolutionary history of aspartic proteases across vertebrates. Notable findings include: (i) the origins of Pgs and *ctse* trace back to the common ancestor of jawed vertebrates; (ii) *ctse* has been completely lost in teleosts; and (iii) *PAG* genes underwent rapid diversification in Cetartiodactyla. These evolutionary patterns correlate closely with major physiological and morphological innovations, such as the acquisition of jaws, the presence or absence of a stomach, specialized feeding strategies (e.g., rumination and hematophagy), and lineage-specific placental morphologies. The evolutionary framework established here provides an essential foundation for deeper insights such as changes in physiological roles and regulatory diversification of gene expression. Integrating molecular evolutionary perspectives with physiological and biochemical evidence will further elucidate how digestive enzyme gene families have contributed to the adaptive evolution of vertebrates.

## Supplementary Information

Supplementary Information 1.

Supplementary Information 2.

Supplementary Information 3.

Supplementary Information 4.

## Data Availability

All datasets used in this study—including gene sequences, whole-genome assemblies, and their accession numbers—are openly available in the Supplemental Information.
