# Supplementary material for: Molecular evolution of aspartic protease gene family in vertebrates
Source: Sci Rep. 2026 May 20;16:22893. doi: 10.1038/s41598-026-52723-0 (PMC13389413; doi:10.1038/s41598-026-52723-0)
Supplement: Supplementary file 4 — Supplementary Information 4. [file 41598_2026_52723_MOESM4_ESM.docx]

**Supplementary Figure 1. Full phylogenetic analysis of all aspartic proteases in vertebrates.**

Maximum-likelihood tree shown in Fig. 1 with all bootstrap values.

**Supplementary Figure 2. Genomic synteny of cathepsin D (*ctsd*).**

Genomic synteny is shown as in Fig. 3, with *ctsd* indicated in orange (“D”).

**Supplementary Figure 3. Genomic synteny of napsin A (*napsa*).**

Genomic synteny of *napsa* genes in (A) tetrapods and ray-finned fishes, and (B) amphibians. *napsa* is shown in bright yellow (“nap”).

**Supplementary Figure 4. Genomic synteny of Beta-Secretase 1 (*bace1*).**

Genomic synteny is shown as in Fig. 3, with *bace1* indicated in gray (“Bs1”).

**Supplementary Figure 5. Genomic synteny of Beta-Secretase 2 (*bace2*).**

Genomic synteny is shown as in Fig. 3, with *bace2* indicated in gray (“Bs2”).

**Supplementary Figure 6. Genomic synteny of nothepsin (*nots*).**

Genomic synteny is shown as in Fig. 3, with *nots* indicated in yellow (“N”).

**Supplementary Figure 7. Genomic synteny of renin (*ren*).**

Genomic synteny is shown as in Fig. 3, with *ren* indicated in light blue (“R”).

**Supplementary Figure 8. Genomic synteny of pepsinogens in teleosts.**

Genomic synteny of (A) pepsinogen C: *pgc* and (B) pepsinogen A: *pga* in teleosts. *pgc* and *pga* are indicated in blue (“C”) and magenta (“A”), respectively.

**Supplementary Figure 9.** **Fully expanded maximum-likelihood phylogenetic tree of all ctse and pepsinogen genes used in this study.**

This figure represents the complete version of Fig. 2, with all terminal sequences and labels shown. Bootstrap support values (ranging from 0 to 1) are indicated at nodes. The tree is shown at high resolution to allow visualization of all labels.

**Supplementary Figure 10. Pseudogenization of cathepsin E (*ctse*) in Cetartiodactyla.**

Pseudogene fragments in (A) exon 5, (B) exon 6, (C) exon 7, (D) exon 8, and (E) exon 9. Each exon was aligned with intact horse *ctse*, and translated amino acid sequences are shown below. Premature stop codons (asterisks) and frameshifts (“X”) are indicated by red circles.

**Supplementary Figure 11. Molecular phylogeny and genomic synteny of teleost cathepsin E (*ctse*).**

(A) Phylogenetic analysis and (B) genomic synteny of teleost *ctse* genes previously identified by previous study^37^. Genes previously identified as teleost *ctse* are highlighted with a black background. Both phylogenetic and synteny analyses strongly support that these genes correspond to nothepsin (*nots*).

**Supplementary Table 1. List of all sequences analyzed in this study**

This table provides a comprehensive list of all species included in this study (common and scientific names), together with the accession numbers of the genome assemblies used and the corresponding genome publication DOIs. For each identified aspartic protease gene, both nucleotide and amino acid sequences are listed along with their accession numbers. Sequences newly predicted in this study are indicated as “in this study”.

**Supplementary Table 2. Genomic regions and coordinates used for synteny comparisons**

This table summarizes the genomic regions examined for comparative synteny analyses. For each species and locus, the genomic coordinates of the analyzed interval are provided, including the flanking genes that define the syntenic block and the physical distance between them.

**Supplementary Table 3. Query sequences used for BLAST searches**

This table lists the initial query sequences used for BLAST-based gene identification. Sequences retrieved from these searches were subsequently used as additional queries in iterative BLAST analyses to improve detection sensitivity. Final gene annotations were determined through integration of molecular phylogenetic analyses and comparative genomic synteny assessments.
